# CD8+ and CD8− NK Cells and Immune Checkpoint Networks in Peripheral Blood During Healthy Pregnancy

**DOI:** 10.3390/ijms26010428

**Published:** 2025-01-06

**Authors:** Matyas Meggyes, David U. Nagy, Livia Mezosi, Beata Polgar, Laszlo Szereday

**Affiliations:** 1 Department of Medical Microbiology and Immunology, Medical School, University of Pecs, 12 Szigeti Street, 7624 Pecs, Hungary; 2 Janos Szentagothai Research Centre, 20 Ifjusag Street, 7624 Pecs, Hungary; 3 Institute of Geobotany/Plant Ecology, Martin-Luther-University, Große Steinstraße 79/80, D-06108 Halle, Germany

**Keywords:** NK cells, CD8, pregnancy, trimester, immune checkpoint pathways

## Abstract

Pregnancy involves significant immunological changes to support fetal development while protecting the mother from infections. A growing body of evidence supports the importance of immune checkpoint pathways, especially at the maternal–fetal interface, although limited information is available about the peripheral expression of these molecules by CD8+ and CD8− NK cell subsets during the trimesters of pregnancy. Understanding the dynamics of these immune cells and their checkpoint pathways is crucial for elucidating their roles in pregnancy maintenance and potential complications. This study aims to investigate the peripheral expression and functional characteristics of CD8+ and CD8− NK cell subsets throughout pregnancy, providing insights into their contributions to maternal and fetal health. A total of 34 healthy women were enrolled from the first, 30 from the second and 40 from the third trimester of pregnancy. At the same time, 35 healthy age-matched non-pregnant women formed the control group. From peripheral blood, mononuclear cells were separated and stored at −80 °C. CD8+ and CD8− NK cell subsets were analyzed from freshly thawed samples, and surface and intracellular staining was performed using flow cytometric analyses. The proportions of CD56+ NK cells in peripheral blood were similar across groups. While CD8− NKdim cells increased significantly in all trimesters compared to non-pregnant controls, CD8+ NKdim cells showed no significant changes. CD8− NKbright cells had higher frequencies throughout pregnancy, whereas CD8+ NKbright cells significantly increased only in the first and second trimesters. The expression levels of immune checkpoint molecules, such as PD-1 and PD-L1, and cytotoxic-activity-related molecules were stable, with notable perforin and granzyme B increases in CD8− NKbright cells throughout pregnancy. Our study shows that peripheral NK cell populations, especially CD8− subsets, are predominant during pregnancy. This shift suggests a crucial role for CD8− NK cells in balancing maternal immune tolerance and surveillance. The stable expression of immune checkpoint molecules indicates that other regulatory mechanisms may be at work. These findings enhance our understanding of peripheral immune dynamics in pregnancy and suggest that targeting CD8− NKbright cell functions could help manage pregnancy-related immune complications. This research elucidates the stable distribution and functional characteristics of peripheral NK cells during pregnancy, with CD8− subsets being more prevalent. The increased activity of CD8− NKbright cells suggests their critical role in maintaining immune surveillance. Our findings provide a basis for future studies to uncover the mechanisms regulating NK cell function in pregnancy, potentially leading to new treatments for immune-related pregnancy complications.

## 1. Introduction

Pregnancy represents a unique immunological state requiring significant adaptations in the maternal immune system to support the semi-allogeneic fetus, highlighting the mystery of how the mother’s immune system tolerates the genetically distinct fetus without rejecting it [1]. The mother’s immune system must adapt during a healthy pregnancy to support adequate fetal development and to protect the mother from infection [2]. Understanding the immunological mechanisms that underlie successful pregnancy outcomes is important, as disruptions in immune regulation can lead to several complications.

To create an immunotolerant environment during pregnancy, a series of immunological modifications occur at the maternal–fetal interface and systemically [3]. These changes involve alterations in both innate and adaptive immune responses, including shifts in the balance of immune cell subsets, changes in cytokine profiles, and the modulation of immune checkpoints and regulatory pathways [3,4,5,6]. Understanding these immunological adaptations is essential for unraveling the mystery of healthy pregnancy.

Among the key players in immune regulation are immune checkpoint molecules, such as T-cell immunoglobulin and mucin-domain containing-3 (TIM-3), programmed cell death protein 1 (PD-1), cytotoxic T-lymphocyte-associated protein 4 (CTLA-4), T-cell immunoglobulin and ITIM domain (TIGIT), CD226 (DNAM-1) and lymphocyte activation gene 3 (LAG-3). These molecules modulate immune activation or suppression by engaging with their corresponding ligands [7,8,9,10]. While CTLA-4 and PD-1 are established functional suppressors, their relevance in NK cell biology remains debated [11,12]. The signaling networks underlying NK cell inhibition are complex, involving interactions between T-cell markers and NK cell regulators, such as TIM-3, TIGIT, CD226 and LAG-3 [7,8,9,13].

Natural killer (NK) cells are traditionally viewed as innate immune cells that bridge innate and adaptive immunity [14,15,16]. During pregnancy, NK cells are particularly enriched in the uterine environment, forming a unique subset known as decidual NK (dNK) cells. These cells exhibit unique phenotypic and functional characteristics compared to peripheral NK (pNK) cells. A substantial proportion of dNK cells derive from the recruitment of peripheral blood CD56brightCD16− and CD56dimCD16+ NK cells [17,18]. dNK cells, abundant in early pregnancy, are crucial for successful placentation, tissue remodeling, vascularization, fetal development and immune tolerance [19,20,21,22,23].

PNK cells constitute 5–10% of peripheral blood lymphocytes in peripheral blood. Over 90% of pNK cells express low-density CD56 (NKdim cells), while approximately 10% of pNK cells have a high-density surface expression of CD56 (NKbright cells). pNKdim cells are predominantly cytotoxic, while pNKbright cells have an immunoregulatory function [24]. About 30% of pNK cells express the CD8α homodimer, and these cells show enhanced survival during target cell killing [25,26]. The presence of CD8 on NK cells suggests a potential overlap with cytotoxic T-cells, and CD8+ NK cells have been observed in various contexts, including viral infections, autoimmune diseases, and cancer [27,28,29]. The precise role of CD8 expression on NK cells is an active area of research.

Deficiencies or dysregulations in NK cell function and phenotype have been associated with adverse pregnancy outcomes. An imbalance or abnormal function of NK cells can lead to pregnancy complications such as recurrent spontaneous abortion, preeclampsia, endometriosis and infertility [30,31,32]. Alterations in NK cell activity have been linked to adverse pregnancy outcomes through mechanisms such as insufficient trophoblast invasion or excessive immune activation, which can disrupt the maternal–fetal interface. Regarding diagnosis and treatment, monitoring NK cell activity can be a useful diagnostic tool for predicting and managing pregnancy complications. For instance, elevated levels of peripheral blood NK cells and their cytotoxic activity have been associated with recurrent spontaneous abortion and implantation failures [24,33]. Treatments such as intravenous immunoglobulin (IVIG) have been shown to reduce elevated NK cell levels, correlating with improved pregnancy outcomes [34,35,36]. Additionally, identifying specific NK cell subsets that promote immune tolerance and fetal growth offers the potential for new immunotherapeutic strategies to assist patients with recurrent miscarriages.

Immune checkpoints are gaining recognition for their potential applications in reproductive immunology. While they are predominantly known for their role in cancer therapy, ongoing research suggests they hold promise for future applications in managing pregnancy-related complications and improving maternal–fetal health. Therefore, understanding NK cell dynamics during pregnancy is critical for identifying potential therapeutic targets and improving maternal–fetal health.

Numerous studies have examined the possible role of different immune checkpoint molecules in healthy or pathological pregnancies. There are no available data about the expression pattern of these molecules by CD8+ and CD8− NK cell subsets in the periphery during the trimesters of human pregnancy. We aimed to investigate these peripheral immune checkpoint pathways and their possible relation with cytotoxic-activity-related molecules throughout the three trimesters of a healthy human pregnancy and in non-pregnant conditions.

## 2. Results

### 2.1. Phenotypic Characteristics of NK Subsets in Peripheral Blood of Healthy Pregnant Women During Each Trimester of Pregnancy and Non-Pregnant Women

**Figure 1:**
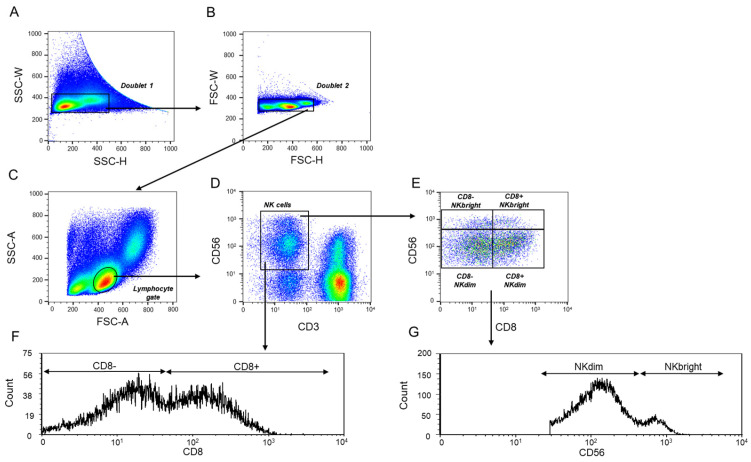
Differentiation of the NK subpopulations using flow cytometric analyses. A flow cytometric gating strategy selected the investigated peripheral CD8+ and CD8− NKdim and NKbright immune cell subpopulations. After a doublet exclusion (**A**,**B**), the lymphocyte gate was created using FSC-A/SSC-A parameters (**C**). The NK cell population was gated from the lymphocyte gate based on the CD3−/CD56+ combination (**D**). Based on the density of the CD56 receptor, the NKdim and NKbright subpopulations were differentiated from the NK cell gate (**E**,**G**). From the NK cell subsets based on the presence of the CD8 receptor (**F**), further subpopulations were differentiated.

Using a flow cytometric gating strategy, following doublet exclusion (Figure 1A,B), NK cells were selected from the lymphocyte gate (Figure 1C) and further divided into NKdim and NKbright subsets based on the expression of the CD56 marker (Figure 1E,G).

The frequencies of CD56+ natural killer (NK) cells within the total lymphocyte population in the peripheral blood of the cohorts did not differ significantly between pregnant and non-pregnant groups (Table 1). This indicates a stable distribution of CD56+ NK cells across different trimesters and non-pregnant states, suggesting that pregnancy does not significantly alter the overall proportion of these cells in peripheral blood.

NKdim and NKbright cell frequencies in the lymphogate and the NK cell gate (Table 1) did not significantly change during the first, second and third trimesters, even compared to non-pregnant women. The frequency of NKdim cells throughout pregnancy was lower and the frequency of NKbright cells was higher than in non-pregnant women, but these results did not reach the significance level (Table 1).

**Figure 2:**
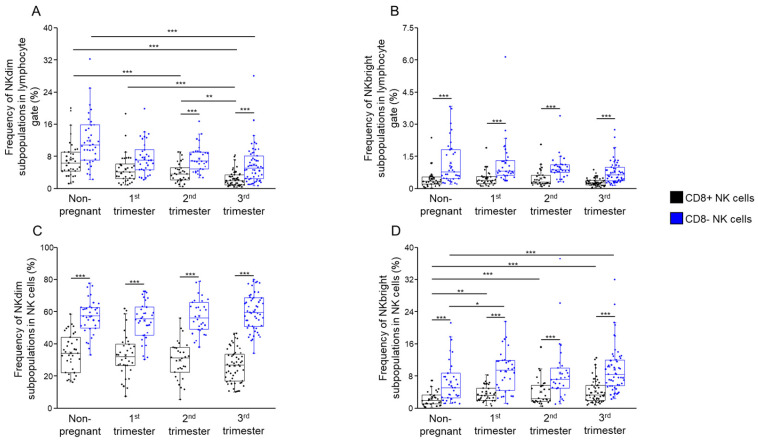
Frequency of NKdim and NKbright subpopulations throughout healthy pregnancy and in non-pregnant women. Frequency of the CD8-receptor-positive and CD8-receptor-negative NKdim and NKbright cells in all lymphocytes (**A**,**B**) and in the NK subpopulation (**C**,**D**) in the three trimesters of healthy pregnancy and healthy, non-pregnant women. The solid bars represent medians of 35, 34, 30 and 40 determinations, the boxes indicate the interquartile ranges and the whiskers represent the variability of the minimum, maximum and any outlier data points in comparison to the interquartile range Significant differences with *p*-Values < 0.01 ***, <0.03 **, <0.05 * are indicated.

Distinct subpopulations of NKdim and NKbright cells were identified based on CD8 surface markers in the peripheral blood of healthy pregnant women during the three trimesters of pregnancy and in healthy non-pregnant women (Figure 1E,G). An analysis of CD56 expression by peripheral blood NK cells revealed that both NKdim and NKbright cells contain CD8+ and CD8− populations. The percentage of CD8− NKdim cells in the lymphocyte gate was significantly higher during the second and third trimesters than that of CD8+ NKdim cells (Figure 2A). When the proportion of the CD8− and CD8+ NKdim cell populations was analyzed in the NKdim cell population, the CD8− NKdim cells showed a significant increase in non-pregnant controls and women in all trimesters than the CD8+ NKdim cells (Figure 2C). Analyzing the differences between the cohorts, we discovered a significant decrease in the frequency of CD8+ NKdim cells in the third trimester compared to non-pregnant women and women in the second and third trimesters.

In the case of NKbright cells, the frequency of CD8− NKbright cells in both the lymphocyte gate and the NKdim cell population was significantly higher in all cohorts compared to the CD8+ counterparts (Figure 2B,D). The CD8+ NKbright cells showed a significant increase in all trimesters compared to non-pregnant women; however, a similar significant elevation was found in the case of CD8− NKbright cells only in the first and second trimesters compared to non-pregnant controls (Figure 2D).

The trend of lower NKdim and higher NKbright cell frequencies during pregnancy may suggest a shift toward a more regulatory immune phenotype crucial for fetal tolerance.

### 2.2. TIGIT and CD226 Receptors Expression by CD8+ and CD8− NK Cells Throughout Healthy Pregnancy and in Non-Pregnant Women

**Figure 3:**
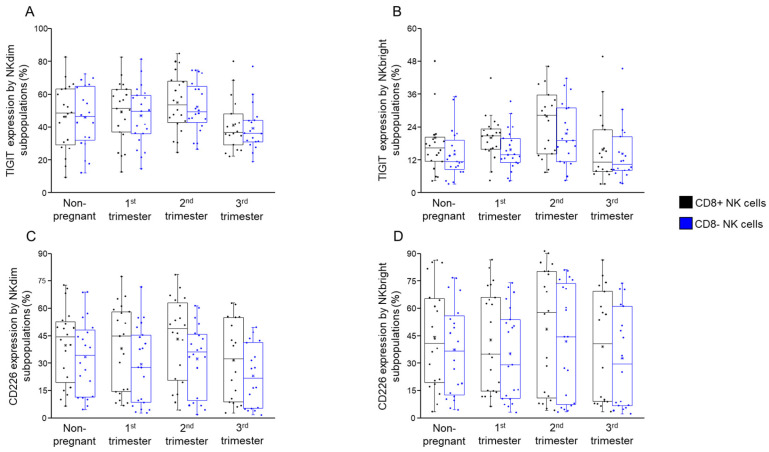
TIGIT and CD226 expression by NKdim and NKbright subpopulations throughout healthy pregnancy and in non-pregnant women. The expression of TIGIT (**A**,**B**) and CD226 receptors (**C**,**D**) by CD8-receptor-positive and CD8-receptor-negative NKdim and NKbright cells during the three trimesters of healthy pregnancy, as well as in healthy non-pregnant women. The solid bars represent medians of 20, 20, 20, and 20 determinations, the boxes indicate the interquartile ranges and the whiskers represent the variability of the minimum, maximum and any outlier data points in comparison to the interquartile range.

No statistically significant differences were observed in the expression levels of TIGIT (Figure 3A,B) or CD226 (Figure 3C,D) between CD8+ NKdim and NKbright cells and their CD8− counterparts in any of the studied groups.

**Figure 4:**
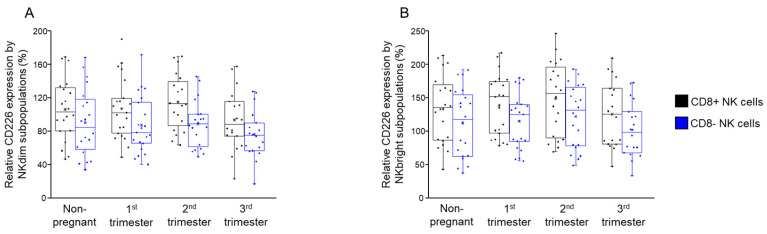
Relative CD226 expression by NKdim and NKbright subpopulations throughout healthy pregnancy and in non-pregnant women. Mean fluorescent intensity (MFI) of the CD226 receptors by the CD8-receptor-positive and negative NKdim (**A**) and NKbright (**B**) cell subpopulations during the three trimesters of healthy pregnancy and in healthy non-pregnant women. The solid bars represent medians of 20, 21, 20 and 18 determinations, the boxes indicate the interquartile ranges and the whiskers represent the variability of the minimum, maximum and any outlier data points in comparison to the interquartile range.

We further analyzed the relative expression of CD226 and TIGIT, as measured by mean fluorescent intensity (MFI), by subpopulations of NK cells categorized based on the presence of the CD8 receptor. Following the analysis, we observed no significant differences among the investigated groups (Figure 4A,B).

**Figure 5:**
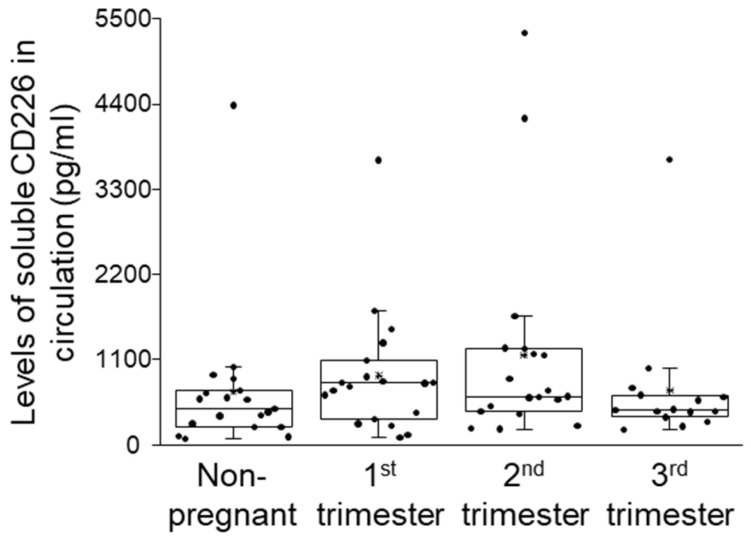
Soluble CD226 levels throughout healthy pregnancy and in non-pregnant women. The serum concentration of the sCD226 molecule in the four investigated cohorts. The solid bars represent medians of 20, 21, 20 and 18 determinations, the boxes indicate the interquartile ranges and the whiskers represent the variability of the minimum, maximum and any outlier data points in comparison to the interquartile range.

No statistically significant difference was observed when the serum levels of the soluble CD226 molecule were compared among the different groups (Figure 5).

**Figure 6:**
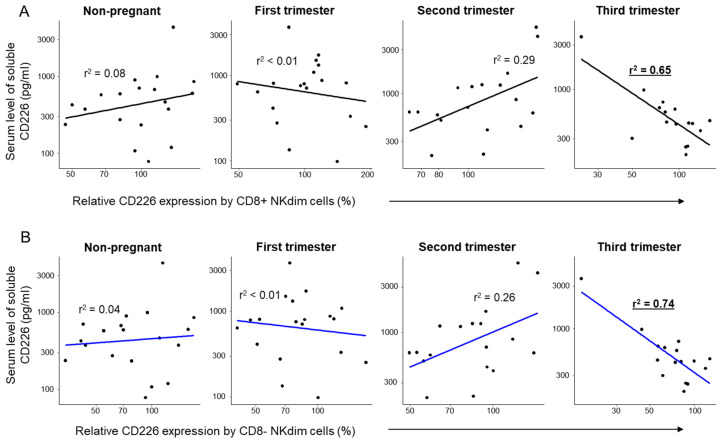
Regression analyses between the relative expression of CD226 and the soluble level of CD226 in NKdim subpopulations throughout healthy pregnancy and in non-pregnant women. Linear regression analyses between the relative expression of CD226 and sCD226 levels in CD8-positive (**A**) and CD8-negative (**B**) NKdim subsets during the three trimesters of healthy pregnancy and in healthy non-pregnant women. *p*-values and coefficients of determination (r^2^) were calculated in R.

**Figure 7:**
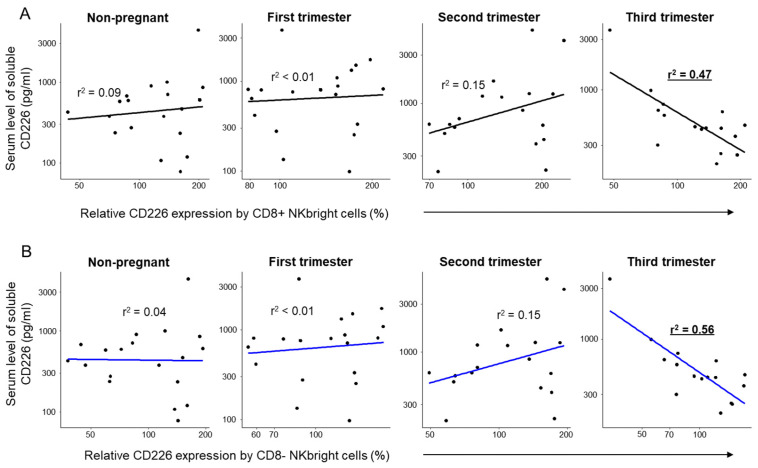
Regression analyses between the relative expression of CD226 and the soluble level of CD226 in NKbright subpopulations throughout healthy pregnancy and in non-pregnant women. Linear regression analyses between the relative expression of CD226 and sCD226 levels in CD8-positive (**A**) and CD8-negative (**B**) NKbright subsets during the three trimesters of healthy pregnancy and in healthy non-pregnant women. *p*-values and coefficients of determination (r^2^) were calculated in R.

Performing a regression analysis to examine the relationship between the serum levels of the soluble CD226 molecule and the relative CD226 expression, a statistically significant negative relationship was observed within both the CD8+ and CD8− NKdim and NKbright subpopulations, specifically limited to the third trimester of pregnancy (Figure 6 and Figure 7).

The stable expression of TIGIT and CD226 throughout pregnancy indicates that these receptors may not significantly influence NK cell function during this period.

### 2.3. CD112 and CD155 Ligand Expression by CD8+ and CD8− NK Cells Throughout Healthy Pregnancy and in Non-Pregnant Women

**Figure 8:**
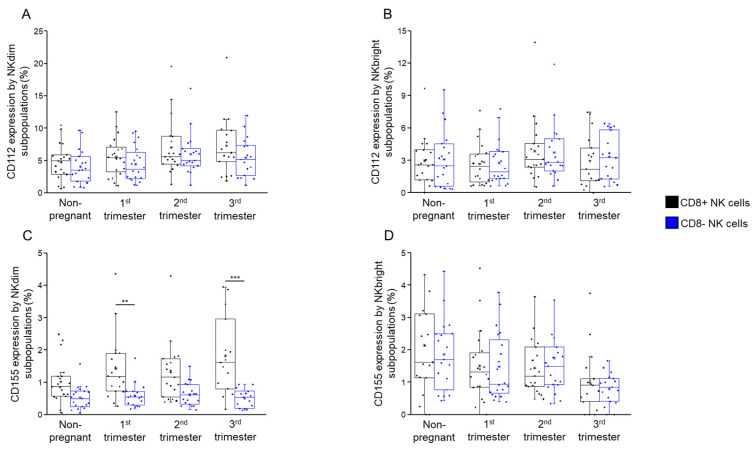
CD112 and CD115 expression by NKdim and NKbright subpopulations throughout healthy pregnancy and in non-pregnant women. The expression of CD112 (**A**,**B**) and CD155 ligands (**C**,**D**) by CD8-receptor-positive and CD8-receptor-negative NKdim and NKbright cells during the three trimesters of healthy pregnancy and in healthy non-pregnant women. The solid bars represent medians of 20, 20, 20 and 17 determinations, the boxes indicate the interquartile ranges and the whiskers represent the variability of the minimum, maximum and any outlier data points in comparison to the interquartile range. Statistically significant differences with *p*-values < 0.03 ** and <0.01 *** are indicated.

No significant differences were observed in the expression levels of CD112 (Figure 8A,B) between CD8+ NKdim and NKbright cells and their CD8− counterparts within any of the studied groups. Through our investigation of CD155 ligand presence, we detected a significant reduction in expression within CD8− NKdim cells when compared to its CD8+ counterpart during the first and third trimesters of a healthy pregnancy (Figure 8C). However, no significant difference was observed when comparing the NKbright subsets (Figure 8D).

The reduction in CD155 expression in CD8− NKdim cells during the first and third trimesters suggests a possible role in enhancing regulatory functions for fetal tolerance.

### 2.4. LAG-3 and Gal-3 Expression by CD8+ and CD8− NK Cells Throughout Pregnancy and in Non-Pregnant Women

**Figure 9:**
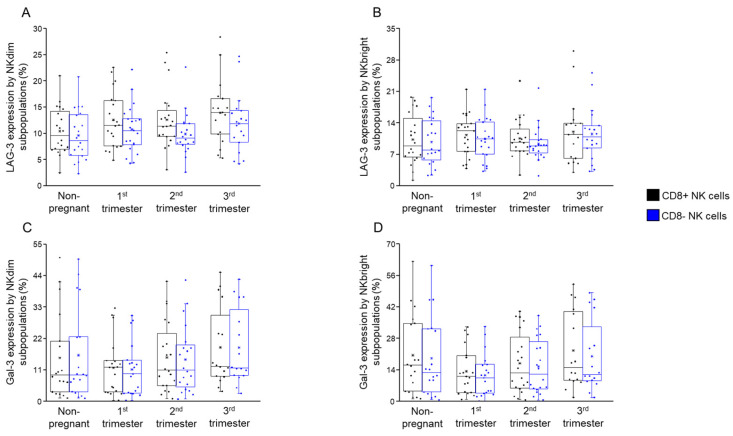
LAG-3 and Galectin-3 expression by NKdim and NKbright subpopulations throughout healthy pregnancy and in non-pregnant women. The expression of LAG-3 (**A**,**B**) and Galectin-3 receptors (**C**,**D**) by CD8-receptor-positive and CD8-receptor-negative NKdim and NKbright cells during the three trimesters of healthy pregnancy and in healthy non-pregnant women. The solid bars represent medians of 20, 20, 20 and 18 determinations, the boxes indicate the interquartile ranges, the whiskers represent the variability of the minimum, maximum and any outlier data points in comparison to the interquartile range.

Neither LAG-3 nor Gal-3 expression by CD8+ NKdim and NKbright subsets and their CD8− counterparts showed a significant difference in any investigated groups (Figure 9A–D).

The consistent expression levels of LAG-3 and Gal-3 imply stable inhibitory regulation of NK cells, which is crucial for maintaining immune balance during pregnancy.

### 2.5. PD-1 and PD-L1 Expression by CD8+ and CD8− NK Cells Throughout Pregnancy and in Non-Pregnant Women

**Figure 10:**
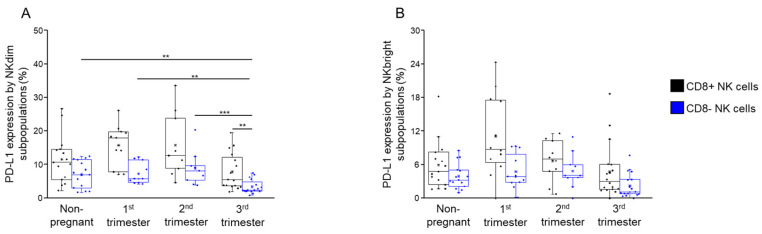
PD-L1 expression by NKdim and NKbright subpopulations throughout healthy pregnancy and in non-pregnant women. The expression of PD-L1 ligand (**A**,**B**) by CD8-receptor-positive and CD8-receptor-negative NKdim and NKbright cells during the three trimesters of healthy pregnancy and in healthy non-pregnant women. The solid bars represent medians of 15, 12, 10 and 16 determinations, the boxes indicate the interquartile ranges and the whiskers represent the variability of the minimum, maximum and any outlier data points in comparison to the interquartile range. Statistically significant differences with *p*-values < 0.03 ** and <0.01 *** are indicated.

Since we could not detect any PD-1 positivity on any NK cell subsets in the peripheral blood of healthy pregnant individuals [37], we measured the surface expression of PD-L1 on NKdim and NKbright cells by flow cytometry. A significant decrease in PD-L1 expression was observed by the CD8− NKdim subset when compared to the CD8+ NKdim cells, but only in women from the third trimester (Figure 10A). Additionally, when comparing the results between the cohorts, it was found that the PD-L1 expression by CD8− NKdim cells mentioned above was significantly lower compared to the other investigated cohort (Figure 10A). A significant difference regarding PD-L1 expression by NKbright subpopulations was not observed (Figure 10B).

The significant decrease in PD-L1 expression by CD8− NKdim cells in the third trimester may enhance activation potential, balancing immune surveillance and fetal protection.

### 2.6. NKG2D Expression by CD8+ and CD8− NK Cells Throughout Pregnancy and in Non-Pregnant Women

**Figure 11:**
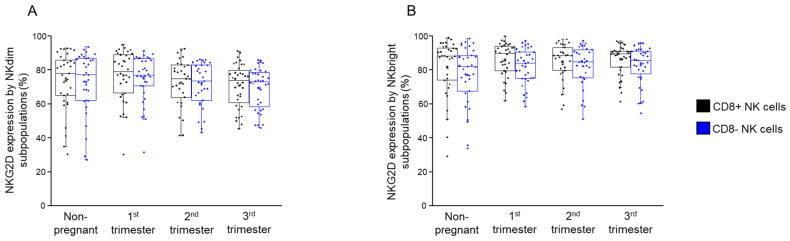
NKG2D expression by NKdim and NKbright subpopulations throughout healthy pregnancy and in non-pregnant women. The expression of NKG2D receptor (**A**,**B**) by CD8-receptor-positive and CD8-receptor-negative NKdim and NKbright cells during the three trimesters of healthy pregnancy and in healthy non-pregnant women. The solid bars represent medians 33, 34, 31 and 36 determinations, the boxes indicate the interquartile ranges and the whiskers represent the variability of the minimum, maximum and any outlier data points in comparison to the interquartile range.

Most (>80%) of the NKdim and NKbright cells expressed NKG2D; hence, this potent activating receptor is expressed by both CD8+ and CD8− NK cells. There was no significant difference in the expression of NKG2D between CD8+ NK subsets and CD8− counterparts in any of the investigated groups (Figure 11A,B).

Stable NKG2D expression across pregnancy stages suggests a maintained readiness of NK cells to respond to stress signals, balancing protection and tolerance.

### 2.7. Cytotoxic Potential of Peripheral CD8+ and CD8− NK Cells Throughout Pregnancy and Non-Pregnant Women

In addition to analyzing the expression of inhibitory and stimulatory immune checkpoint receptors and their corresponding ligands, the effector functions of these cells were investigated.

Flow cytometric analysis of cytotoxicity-related molecules suggested a similar cytotoxic capacity of CD8+ and CD8− NKdim cells. Most CD8+ and CD8− NKdim cells expressed perforin and granzyme B, which are important effectors of NK cell cytotoxicity.

**Figure 12:**
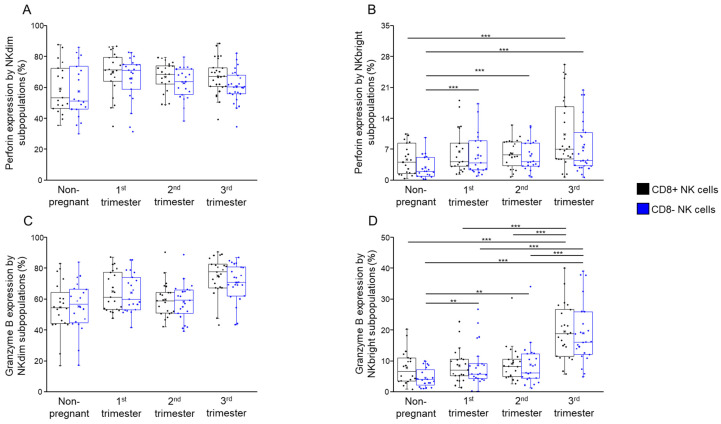
Perforin and granzyme B content by NKdim and NKbright subpopulations throughout healthy pregnancy and in non-pregnant women. The expression of intracellular perforin (**A**,**B**) and granzyme B (**C**,**D**) by CD8-receptor-positive and CD8-receptor-negative NKdim and NKbright cells during the three trimesters of healthy pregnancy and in healthy non-pregnant women. The solid bars represent medians of 19, 21, 21 and 26 determinations, the boxes indicate the interquartile ranges and the whiskers represent the variability of the minimum, maximum and any outlier data points in comparison to the interquartile range. Statistically significant differences with *p*-values < 0.03 ** and <0.01 *** are indicated.

However, no statistical difference was revealed regarding the NKdim subpopulations (Figure 12A,C), and the expression of perforin by CD8− NKbright cells was found to be significantly increased throughout pregnancy compared to the non-pregnant state (Figure 12B). Conversely, CD8+ NKbright cells exhibited higher perforin expression only during the third trimester when compared to non-pregnant women (Figure 12B). On the other hand, CD8+ NKbright cells exhibited a significant increase in granzyme B expression only during the third trimester of healthy pregnancy compared to non-pregnant controls (Figure 12D).

At the same time, granzyme B content was measured and found to be significantly elevated in the CD8− NKbright subpopulation throughout pregnancy compared to that in non-pregnancy. Specifically, granzyme B expression by CD8− NKbright cells was significantly higher in the third trimester than in the first and second trimesters. On the other hand, the expression of granzyme B by CD8+ NKbright cells was significantly elevated only in the third trimester during healthy pregnancy compared to the first and second trimesters and non-pregnant states (Figure 12D).

The increased cytotoxic activity by CD8− NKbright cells throughout pregnancy highlights their role in maintaining immune surveillance during this period.

## 3. Discussion

The maternal immune system undergoes a complex transformation as early as the beginning of pregnancy. These profound changes protect the fetus from a harmful immune response. It was once believed that the immune response during pregnancy was primarily adaptive in early pregnancy and maintained throughout gestation. However, three immunological phases occur during a healthy pregnancy [38,39]. The initial phase, observed predominantly during the first trimester, is characterized by a strong inflammatory response. This inflammatory environment is mandatory in order to ensure an adequate reconstruction of the uterine epithelium, the elimination of cellular debris, and tissue remodeling. The next immunological phase of pregnancy is an anti-inflammatory state. The hallmark of this second phase (second and third trimesters) is rapid fetal growth and development [40]. The last immunological stage occurs late in the pregnancy once fetal development is complete and the mother is prepared to deliver the baby. This is achieved through a renewed inflammatory response [41]. Hormonal fluctuations play a pivotal role in regulating these immunological phases, with hormones such as progesterone, estrogen and human chorionic gonadotropin (hCG) acting as critical modulators of immune tolerance and function during pregnancy [42]. The immunology behind birth is very complex, contradictory and, in part, poorly understood. The local environment at the time of labor is inflammatory, which is detectable in maternal immune cells, including the activation of neutrophils, increased frequencies of NK cells and increased inflammatory cytokines [43,44].

The interplay between inflammatory markers and immune cell populations is particularly significant during pregnancy, as inflammation fluctuates across its distinct immunological phases [6,38,40]. During the third trimester, a renewed inflammatory response is often observed, characterized by elevated levels of inflammatory cytokines, acute-phase proteins and increased neutrophil activity [41]. These inflammatory changes are thought to influence immune cell dynamics, including alterations in lymphocyte populations and activation states of NK cells [6,45]. For instance, C-reactive protein (CRP) and other acute-phase proteins may serve as systemic indicators of this inflammatory environment, correlating with changes in NK cell distribution and function [46]. Investigating these parameters in conjunction with immune cell profiles could provide deeper insights into the mechanisms governing maternal–fetal immune balance.

The frequencies of NK cell subsets are closely associated with inflammatory markers, reflecting their dynamic role in immune modulation. During a healthy pregnancy, notable shifts in NK cell frequency and phenotype are observed [47]. Specifically, the population of NKbright cells increases progressively across the trimesters, peaking in the third trimester, while a reciprocal decline in the cytotoxic NKdim cell subset occurs [47]. NKbright cells are often reduced in conditions of chronic inflammation, as evidenced by their negative correlation with inflammatory markers like CRP [48]. Another study notes that the regulatory NKbright subset positively correlates with IL-12p40 and MCP-1 while negatively correlating with IP-10 and TNF-α, suggesting a potential protective function against excessive inflammation [49]. These findings underscore the dynamic interplay between NK cell dynamics and systemic inflammation, providing valuable insights into immune regulation in both physiological and pathological conditions.

The balance between fetal and maternal adaptive immune systems is also crucial in term and preterm labor [50]. Progesterone, in particular, has been shown to suppress NK cell cytotoxicity by altering cytokine production and receptor expression [51]. This hormonal influence may contribute to the distinct NK cell profiles observed in our study, particularly during the late stages of pregnancy.

Over the past two decades, significant progress has been made in understanding the biological properties of NK cells, highlighting their crucial role in various fields of medicine [52]. In oncology, NK cells are recognized as potent anti-tumor agents, positioning them as a cornerstone in developing innovative cancer treatments [53]. Beyond oncology, NK cells have shown promise in infectious disease control, where their ability to target and destroy virally infected cells is invaluable [54]. Additionally, NK cells play a critical role in immunoregulation and transplantation, contributing to graft acceptance and minimizing rejection [55]. This growing body of research underscores the immense therapeutic potential of NK cells, paving the way for new and effective immunotherapeutic strategies across multiple medical disciplines.

Despite these advancements, the functional significance of CD8 expression by human NK cells has been largely overlooked. This study investigates the distribution of these NK cell subsets in peripheral blood throughout healthy pregnancies and in non-pregnant individuals.

Hormonal fluctuations might influence the migration of CD8+ NK cells to the maternal–fetal interface, a hypothesis supported by evidence showing chemokine receptor expression modulation by pregnancy-associated hormones [56,57]. Immune checkpoint molecules, such as PD1, CTLA4, TIM-3, TIGIT, CD226 and LAG3, have been mainly studied in effector T-cell exhaustion [22]. We investigate their possible role in influencing NK cells in pregnancy progression.

NK cells are essential for successful reproductive outcomes due to their pivotal role throughout pregnancy. These cells contribute to the establishment and maintenance of early pregnancy by promoting the remodeling of uterine blood vessels, ensuring adequate blood flow to the developing fetus. Additionally, NK cells regulate the maternal immune response, preventing the rejection of the fetus by the maternal immune system. As pregnancy advances toward labor, NK cells produce cytokines and chemokines that influence uterine contractions and the rupture of fetal membranes. Therefore, NK cells are critical for sustaining pregnancy and facilitating the processes leading to a safe and effective delivery [20,23]. Consistent with previous studies, we found no significant changes in peripheral blood NK cell levels during pregnancy compared to non-pregnant controls [58,59].

We sought to investigate potential immune mechanisms explaining the association of CD8 positivity on NK cell subpopulations with immune changes during the three trimesters of a healthy pregnancy. In this report, we show that most NK cells of human peripheral blood lack CD8 expression not only in non-pregnant women but also throughout a healthy pregnancy. One possible reason for the selective increase in CD8− NK cells in the peripheral blood could be a preferential migration of CD8+ NK cells into the maternal–fetal interface due to the differential expression of chemokine receptors on the different NK cell subsets [60]. It is also possible that the hormonal microenvironment selectively attracts or retains certain NK cell subsets at the interface.

Importantly, we demonstrate that both CD8− and CD8+ NK cells have similar functional properties during pregnancy in contrast to a previous study by Lindgren et al. showing that CD8− NK cells present in the gastrointestinal mucosa could play a role in the innate defense against gastrointestinal bacterial infections [61].

NK cell activity during maternal immune tolerance is modulated by several factors, such as the cytokine environment; receptor expression on NK cells, including immune checkpoint pathways; and interactions with fetal antigens, progesterone and many others [22,23]. Estrogen has also been shown to regulate NK cell function [62]. This hormonal regulation may provide a broader explanation for the immune balance observed during pregnancy.

We noticed a significant decrease in the frequency of CD8+ NKdim cells in the lymphocyte gate in the third trimester compared to non-pregnant women and women in the second and third trimesters. In contrast, the frequency of CD8+ NKbright cells showed a significant increase in all trimesters compared to non-pregnant women. A similar tendency was found in the case of CD8− NK cells, which reached a level of significance only in the third trimester compared to the non-pregnant state. These observations are consistent with previous studies that reported hormonal changes during pregnancy, particularly in progesterone levels, which modulate NK cell activity and distribution [51,63]. Based on the above observations, we hypothesize that NKdim cells play a role throughout the three trimesters.

As pregnancy progresses, peripheral NKbright cells gain the cytoplasmic granules (perforin and granzyme B) necessary for late pregnancy and parturition; we asked whether an altered immune checkpoint pathway might explain this. Despite prior studies reporting higher cytolytic activity of CD8+ NK cells compared to CD8− NK cells [25,64], our investigation did not reveal any significant difference in cytotoxic potential between these subsets, both in non-pregnant individuals and throughout pregnancy. This contradicts another recent study by Addison et al. [25], which shows that CD8+ NK cells have a higher cytotoxic activity than CD8− NK cells, mediated by the prevention of activation-induced apoptosis by the ligation of CD8α.

A recent study investigated an intriguing association between a decreased surface expression of the CD226 receptor by CD8+ T- and NK cell populations and an elevated CD226 serum level in cutaneous T-cell lymphoma patients [65]. This study suggests that the CD226 surface molecule may undergo shedding from the cell membrane, leading to its entry into the systemic circulation. Based on these findings, we performed regression analyses to examine the association between the soluble CD226 level and the relative CD226 expression in distinct NKdim and NKbright subpopulations. Significantly negative relationships were revealed in all investigated subsets, exclusively confined to the third trimester. These findings shed light on the altered immunological environment near the onset of labor and underscore the importance of the TIGIT/CD226/CD112/CD155 immune checkpoint network in reproductive immunology. Given the significant hormonal changes in the third trimester, it is plausible that these hormones influence the altered immune profile observed during late pregnancy.

Peripheral blood NK lymphocytes could reflect local immune system conditions at the maternal–fetal interface and may be significant for reproduction. We have previously shown that NK cells’ activating receptor expression, activation and cytotoxicity imbalance correlate with IVF failure [66]. Assessing the clinical and immune-phenotypes association of CD8+/CD8− balance on peripheral blood NK population, Dons’koi found that decreased or increased CD8 NK phenotypes are unfavorable for implantation or pregnancy development [67]. Based on the above research, it is unsurprising that none of the examined immune checkpoint molecules (TIGIT, CD226 and LAG-3) showed any significant change during a healthy pregnancy. Investigating the expression profile of the relevant ligands, significant alterations were observed exclusively within the NKdim population in a distinct manner for CD155 and PD-L1. While the expression levels of CD112 and Gal-3 remained unchanged throughout healthy pregnancy, CD155 expression exhibited significant differences based on the presence or absence of CD8 in both the early and late stages of pregnancy. Conversely, PD-L1 expression was significantly reduced, specifically within the CD8− NKdim population during the third trimester, compared to the other trimesters and the non-pregnant state. These findings suggest potential regulatory mechanisms specific to these ligands in modulating immune response in different stages of pregnancy by various immune cell subsets.

Furthermore, published evidence shows that CD8+ and CD8− NK cells might respond differently in certain disease settings. Studies of HIV infection have shown differential responses between these subsets, with CD8+ and CD8− NK subpopulations often demonstrating different cytotoxicity and susceptibility to exhaustion [28,68]. Understanding these nuanced interactions is crucial, as it could lead to the development of targeted therapies or diagnostic tools to improve reproductive outcomes.

Emerging evidence highlights the therapeutic potential of modulating NK cell subsets, particularly decidual NK cells, in pregnancy complications [30,32,69]. CD8+ NK cells, known for their enhanced cytotoxic activity, may have distinct roles at the maternal–fetal interface, driven by differential chemokine receptor expression. Dysregulation of immune checkpoint pathways, like the TIGIT/CD226 axis, has been implicated in complications such as recurrent implantation failure and preeclampsia, suggesting a role for checkpoint modulation in therapeutic strategies [67,70]. Additionally, soluble checkpoint molecules, such as CD226, show promise as targets in managing immune responses [71]. Targeted approaches, including cytokine modulation, enhancing immune checkpoint interactions and incorporating IVIG therapy, could restore NK cell balance and mitigate pregnancy-related complications [69,72,73]. These findings emphasize the need for further investigation into NK cell subsets as therapeutic targets.

Given that the expression of NKG2D by these NK cell subpopulations, regardless of CD8 positivity, showed no difference at all, we conclude that none of the investigated receptors directly mediate the function of either CD8+ or CD8− NK cell subpopulations throughout a healthy pregnancy. This finding underscores the necessity for a deeper understanding of the role of CD8 in human NK cells. Our data lead us to hypothesize that a well-coordinated regulation by other immune checkpoint pathways may control the potentially harmful NK cell populations during a healthy pregnancy. These insights pave the way for future research to unravel the complex regulatory networks governing NK cell function, ultimately contributing to developing targeted therapies that ensure maternal and fetal health during pregnancy.

## 4. Limitations

1. Single-center study: The study was conducted at a single center, which may limit the diversity of the participant population and the generalizability of the findings to other settings or people.
2. Phenotypic focus: The study focused on the phenotypic characterization of NK cells and their expression of immune checkpoint molecules. The functional activity of NK cells, such as cytokine production or cytotoxicity assays, was not assessed. Functional assays could provide a more comprehensive understanding of prenatal NK cell activity.
3. Cross-sectional design: The study followed a cross-sectional design, analyzing NK cell populations at different trimesters of pregnancy. A longitudinal study design would allow for the evaluation of changes within individual participants over time, providing deeper insights into the dynamics of NK cell populations during pregnancy.
4. Limited receptor analysis: Although the study investigated several immune checkpoint molecules, the analysis was limited to a specific set of receptors. Additional immune checkpoint molecules relevant to NK cell biology could have provided a more comprehensive evaluation of their role in pregnancy.
5. Maternal–fetal interface: Exploring NK cells at the maternal–fetal interface could yield valuable insights into their role in pregnancy. However, assessing decidual NK cells during an ongoing pregnancy is inapplicable, as it requires an invasive procedure posing surgical and infectious risks.
6. Focus on NK cells: The study primarily focused on NK cells and did not compare their profiles to those of other immune cell populations involved in pregnancy. Comparing them with T-cells or regulatory immune cells could provide a broader understanding of the immune interactions occurring during pregnancy.
7. Hematological and inflammatory parameters: The study did not include hematological and inflammatory parameters. These data would have provided valuable context for understanding the immune environment during pregnancy and could have offered insights into correlations between NK cell counts and inflammatory status.

## 5. Materials and Methods

### 5.1. Participants

The study included 104 pregnant women, divided into 3 different groups according to their corresponding trimesters—34 healthy pregnant volunteers were recruited from the first trimester, 30 from the second trimester and 40 from the third trimester of pregnancy—in collaboration with the Department of Obstetrics and Gynecology at the University of Pecs (Table 2). Each group consisted of different patients. A total of 35 healthy age-matched non-pregnant women were recruited for the control group.

In accordance with the guidelines set forth by the EU-GDPR and the Privacy Informational and Healthy Data Act Regulations, the handling of health-related and personal information gathered during the blood collection process adhered to stringent scientific protocols. This ensured that the data were processed anonymously, securely and confidentially, with no provision of data to the research team for subsequent demographic analysis.

The health profiles of each participant were meticulously assessed. This comprehensive evaluation confirmed that all participants had no significant medical history, refrained from medication use (including hormonal contraceptives) and had not recently experienced any illnesses. Moreover, women who exhibited complications related to pregnancy, infections, pre-existing medical conditions before conception, pregnancies resulting from in vitro fertilization, immune-related disorders, diabetes mellitus or AIDS were intentionally excluded from the study cohort. None of the participants was a tobacco consumer.

### 5.2. Sample Collection, PBMC Separation and Cryopreservation

Twenty milliliters of venous blood was collected into heparinized tubes and transported to the laboratory for further investigation. Afterward, peripheral blood mononuclear cells (PBMCs) had been separated from heparinized venous blood on Ficoll-Paque density (GE Healthcare, Chicago, IL, USA) gradient. Isolated cells were washed in RPMI 1640 medium (Lonza, Switzerland), counted and centrifuged. PBMCs were then resuspended in human serum containing 10% DMSO (Sigma-Aldrich, St. Louis, MO, USA) for cryoprotection. Next, the cells were aliquoted in cryovials and stored in a −80 °C mechanical freezer. On the day of fluorescent cell labeling, the cryovials were warmed up in a 37 °C water bath and DMSO was washed out twice in RPMI 1640 medium. Cell viability was assessed utilizing trypan blue exclusion prior to the flow cytometric analysis (consistently >90%).

Ten milliliters of venous blood were collected into serum tubes for each participant. Serum samples were aliquoted in cryovials and stored at −80 °C until ELISA examination. The storage and handling protocols ensured sample integrity for subsequent assays.

### 5.3. Flow Cytometric Measurement

For flow cytometric measurement, 10^6^ PBMCs were incubated with fluorochrome-conjugated monoclonal antibodies (Table 3) for 30 min at room temperature (RT) in complete darkness. Afterward, the cells were washed and resuspended in 300 µL phosphate-buffered saline (PBS) (BioSera, Cholet, France) containing 1% paraformaldehyde (PFA) and stored at 4 °C in darkness until flow cytometric analysis. Flow cytometric measurements were performed using a BD FACS Canto II flow cytometer (BD Immunocytometry Systems, Erembodegem, Belgium) with BD FACS Diva V6 Software (BD Biosciences, Franklin Lakes, NJ, USA) used for data acquisition. Flow cytometric data were analyzed by FCS Express V4 (De Novo Software, Pasadena, CA, USA) [74].

### 5.4. Intracellular Staining

Following surface staining, cells were washed with PBS and fixed in 4% PFA for 10 min at RT in complete darkness. After the fixation step, the cells were washed with PBS and permeabilized using a 1:10 dilution of FACS Permeabilizing Solution 2 (BD Biosciences) for 10 min at RT in darkness. Subsequently, the samples were washed with PBS and incubated with FITC-conjugated anti-human granzyme B and PE-Cy7-conjugated anti-human perforin antibodies for 30 min at RT in darkness. Finally, the cells were washed with PBS, fixed with 1% PFA and stored at 4 °C in darkness until further FACS analysis [74].

### 5.5. Enzyme-Linked Immunosorbent Assay (ELISA)

DNAM1 (CD226) concentrations were quantified by sandwich Enzyme-Linked Immunosorbent Assays (ELISA, R&D Systems, Bio-Techne, Minneapolis, MN, USA, DY666-05, Ancillary Reagent kit 2: R&D Systems, Bio-Techne, DY008) following previously published methods [74]. A 96-well microplate was coated overnight with 100 µL/well anti-human CD226 antibody at RT. Subsequently, the plate was washed and blocked with 400 µL/well of Reagent diluent for an hour. After another washing step, serially diluted standards and serum samples (100 µL) were added to the wells and incubated for two hours at RT. Following incubation, the wells were rewashed and incubated with a diluted detection antibody (100 µL) for two hours at RT. After another washing step, the wells were incubated with streptavidin–HRP (100 µ/well) for 20 min. The plate was developed with Substrate Reagents A + B (1:1) after the final wash for 20 min in darkness. The reaction was then terminated using a stop solution. The absorbance of the test plates was measured at 450 nm or 450 nm with a reference filter of 540 nm using a BMG SPECTOstar Nano spectrophotometer (BMG Labtech, Ortenberg, Germany). Standard curves were generated using 4-parametric logistic analysis with background subtraction, and DNAM-1 levels were quantified using MARS Data Analysis Software version 3.32 (BMG Labtech, Ortenberg, Germany).

### 5.6. Data Analysis

For the analysis of the differences between the investigated parameters of NKdim and NKbright subpopulations, linear models were run in R. Decisions on the transformation of response variables depended on a visual inspection of “model-checking plots” for the models with transformed vs. untransformed variables. Based on these plots, the normality of residuals and the assumption of the homogeneity of variance were checked [75]. Variables were log-transformed. Explanatory variables were the two-way interaction effects of cell types (CD8+ or CD8−) × trimester (non-pregnant, first, second and third trimesters). A two-way ANOVA was used to test for statistical significance. For pair-wise comparisons, Tukey post hoc tests were conducted to compare each cell type/status combination to each other.

Additionally, linear regression models were conducted separately for ELISA and CD8 measures across the different trimesters. Each variable was log-transformed.

## 6. Conclusions

This study elucidates the phenotypic and functional profiles of peripheral NK cells during pregnancy, highlighting the predominance of CD8− subsets and the consistent expression of immune checkpoint molecules across trimesters. The findings suggest that the distribution of NK cell subsets remains stable during pregnancy, with CD8− and CD8+ NK cells having similar functional properties. Despite the detailed analysis of specific immune checkpoint molecules, additional research is needed to fully understand their roles in pregnancy and the potential mechanisms regulating NK cell function.

Future research should focus on longitudinal studies to observe the dynamics of NK cell populations over time and functional assays to provide a more comprehensive understanding of NK cell activity during pregnancy. Additionally, exploring the interactions between NK cells and other immune cell populations at the maternal–fetal interface could yield valuable insights into their collective roles in maintaining pregnancy.

Ultimately, our findings lay the groundwork for potential therapeutic interventions to modulate NK cell activity to prevent pregnancy-related complications. By advancing our understanding of the immune mechanisms at play, we can move closer to developing targeted treatments that ensure maternal and fetal health, paving the way for improved outcomes in reproductive medicine.

## Figures and Tables

**Table 1:** Comparison of the frequencies of different NK cell subpopulations throughout pregnancy and in non-pregnant women.

	Gate	Non-Pregnant	1st Trimester	2nd Trimester	3rd Trimester	*p*-Value
NK cell	Lymphocyte	17.83 ± 8.09	13.61 ± 6.87	11.98 ± 5.18	12.75 ± 6.87	NS
NKdim	Lymphocyte	16.46 ± 7.60	12.13 ± 6.53	10.73 ± 5.23	11.51 ± 6.22	NS
NKbright	Lymphocyte	1.53 ± 1.73	1.48 ± 0.88	1.27 ± 0.65	1.58 ± 0.90	NS
NKdim	NK cell	92.18 ± 5.37	87.97 ± 5.96	87.91 ± 8.34	86.74 ± 7.67	NS
NKbright	NK cell	8.22 ± 5.51	11.94 ± 5.82	12.19 ± 8.47	13.54 ± 7.82	NS

Statistical comparisons were made in R using one-way ANOVA tests. The results were presented as the mean value ± SD. Statistical differences among the investigated cohorts were not detected. NS: not significant.

**Table 2:** Gynecological and demographic data of the participating women.

	Non-Pregnant	1st Trimester	2nd Trimester	3rd Trimester	*p*-Value
No. of women	35	34	30	40	NS
Age (years)	30.93 (22–43)	31.22 (18–40)	32.07 (23–46)	32.46 (26–43)	NS
Gestation age at sampling (weeks)	-	13.09 ± 2.27	25.32 ± 1.81	32.37 ± 4.10	NS
Gestation age at birth (weeks)	-	39.00 ± 1.50	39.03 ± 0.98	39.24 ± 0.89	NS
Gravidity	-	0.77	1.00	1.33	NS
Parity	-	0.64	0.93	1.12	NS

Statistical comparisons were made in R using one-way ANOVA tests. Data are shown as the mean value ± standard deviation (SD) of the mean. Statistical differences among the investigated cohorts were not detected. NS: not significant.

**Table 3:** Fluorochrome-conjugated monoclonal antibodies were used in the study.

Antigen	Format	Clone	Isotype	Company	CAT
CD112	PE	R2.525	Mouse IgG1, κ	BD Biosciences	551057
CD155	APC	SKII.4	Mouse IgG1, κ	Biolegend	337618
CD3	BV510	UCHT1	Mouse BALB/c IgG1, κ	BD Biosciences	563109
CD8	APC-H7	SK1	Mouse BALB/c IgG1, κ	BD Biosciences	560179
CD56	APC	B159	Mouse IgG1, κ	BD Biosciences	555518
CD226	BV421	DX11	Mouse BALB/c IgG1, κ	BD Biosciences	742493
Galectin-3	PE	B2C10	Mouse BALB/c IgG1, κ	BD Biosciences	565676
Granzyme B	FITC	GB11	Mouse BALB/c IgG1, κ	BD Biosciences	560211
LAG-3	PerCp Cy5.5	11C3C65	Mouse IgG1, κ	Biolegend	369312
NKG2D	PE-Cy7	1D11	Mouse RBF/DnJ IgG1, κ	BD Biosciences	562365
Perforin	PE-Cy7	dG9	Mouse IgG2b, κ	Biolegend	308126
PD-L1	BV421	MIH1	Mouse BALB/c IgG1, κ	BD Biosciences	563738
TIGIT	PE	A1553G	Mouse IgG2a, κ	Biolegend	372704

## Data Availability

The data used to support the findings of this study are available from the corresponding author upon request.
